# Alcohol induced hepatic retinoid depletion is associated with the induction of multiple retinoid catabolizing cytochrome P450 enzymes

**DOI:** 10.1371/journal.pone.0261675

**Published:** 2022-01-14

**Authors:** Afroza Ferdouse, Rishi R. Agrawal, Madeleine A. Gao, Hongfeng Jiang, William S. Blaner, Robin D. Clugston

**Affiliations:** 1 Department of Physiology, University of Alberta, Edmonton, Alberta, Canada; 2 Institute of Human Nutrition, Columbia University, New York, New York, United States of America; 3 Department of Medicine, Columbia University, New York, New York, United States of America; 4 Key Laboratory of Remodeling-Related Cardiovascular Diseases, Ministry of Education, Beijing Collaborative Innovation Center for Cardiovascular Disorders, Beijing Anzhen Hospital, Capital Medical University, Beijing, China; University of Delaware, UNITED STATES

## Abstract

Chronic alcohol consumption leads to a spectrum of liver disease that is associated with significant global mortality and morbidity. Alcohol is known to deplete hepatic vitamin A content, which has been linked to the pathogenesis of alcoholic liver disease. It has been suggested that induction of Cytochrome P450 2E1 (CYP2E1) contributes to alcohol-induced hepatic vitamin A depletion, but the possible contributions of other retinoid-catabolizing CYPs have not been well studied. The main objective of this study was to better understand alcohol-induced hepatic vitamin A depletion and test the hypothesis that alcohol-induced depletion of hepatic vitamin A is due to CYP-mediated oxidative catabolism. This hypothesis was tested in a mouse model of chronic alcohol consumption, including wild type and *Cyp2e1* ^*-/-*^ mice. Our results show that chronic alcohol consumption is associated with decreased levels of hepatic retinol, retinyl esters, and retinoic acid. Moreover, the depletion of hepatic retinoid is associated with the induction of multiple retinoid catabolizing CYPs, including CYP26A1, and CYP26B1 in alcohol fed wild type mice. In *Cyp2e1* ^*-/-*^ mice, alcohol-induced retinol decline is blunted but retinyl esters undergo a change in their acyl composition and decline upon alcohol exposure like WT mice. In conclusion, the alcohol induced decline in hepatic vitamin A content is associated with increased expression of multiple retinoid-catabolizing CYPs, including the retinoic acid specific hydroxylases CYP26A1 and CYP26B1.

## Introduction

Liver disease accounts for approximately 2 million global deaths per year, the major cause of which is heavy alcohol consumption [1, 2]. As the global burden of liver disease increases, epidemiological studies indicate an emerging interaction between alcohol abuse and obesity in the development and progression of liver disease leading to worse outcomes [3, 4]. A range of liver disease is associated with heavy alcohol consumption, including steatosis, fibrosis, alcoholic hepatitis, and cirrhosis, which are collectively referred to as alcohol-associated liver disease (ALD). Given the increasing incidence and disease burden of ALD, research into its pathogenesis is a global health priority.

Alcohol consumption has been associated with the altered metabolism of vitamin A, which may be an important determinant of ALD pathogenesis [5]. There are three important vitamin A metabolites to consider in this context, retinol, retinyl ester, and retinoic acid (RA). Retinol is derived from the diet and is the precursor molecule for RA synthesis, which is the active metabolite of vitamin A and a potent transcriptional regulator. When dietary vitamin A is in excess, retinol can be esterified to form retinyl ester for storage in hepatic stellate cells (HSCs) [6]. Note, hereafter we use the term retinoid synonymously with vitamin A, using it to refer collectively to the vitamin A metabolites retinyl ester, retinol, and RA. Adults with ALD display decreasing hepatic retinoid levels with increasing severity of hepatic disease [7, 8]. These individuals may have normal serum retinol levels, but their hepatic retinoid content can still be severely depleted [8]. This hepatic retinoid depletion is independent of dietary retinoid intake or malabsorption, as confirmed in animal models chronically consuming ethanol along with nutritionally controlled dietary vitamin A levels [9, 10]. Thus, there appears to be an association between alcohol consumption and the depletion of hepatic retinoid in the development of ALD, but the molecular mechanism underlying this remains unclear. It is thought that alcohol consumption decreases hepatic retinoid levels either by increasing retinoid catabolism within the liver or by increasing its mobilization to extrahepatic tissues, making it less available for the synthesis of RA, the most bioactive form of vitamin A [5, 11, 12]. Indeed, we recently showed that alcohol has a biphasic effect on hepatic retinoid metabolism, characterized by an early mobilization phase followed by cytochrome P450 (CYP) mediated catabolism [13]. Furthermore, while previous studies suggested that alcohol-induced hepatic retinoid depletion may be due to oxidative catabolism of retinol mediated by CYP2E1 [11, 14], our recent study in *Cyp2e1*^-/-^ mice established that although hepatic retinol levels are preserved in alcohol-fed mice, retinyl esters are still depleted [13]. This finding indicated that CYP2E1 may not be the major CYP enzyme responsible for alcohol induced catabolism of hepatic retinoid, as previously hypothesized [11, 14]. The goal of this study was to identify other possible contributors to alcohol-induced hepatic retinoid catabolism. This includes the contribution of the RA-specific hydroxylases CYP26A1 and CYP26B1, as well as other broader-specificity RA-metabolizing CYP enzymes that have not been well-studied in the context of chronic alcohol consumption [15].

To better understand CYP-mediated hepatic retinoid depletion in alcohol fed mice, we hypothesized that alcohol-induced depletion of hepatic retinoid is due to CYP-mediated oxidative catabolism. To test this hypothesis, we evaluated the expression of known retinoid catabolizing CYP enzymes in alcohol-fed mice and conducted a genetic dissection of CYP2E1’s contribution to alcohol-induced hepatic retinoid depletion.

## Materials and methods

### Mice, alcohol feeding protocol, and tissue collection

All animal experiments were approved by Columbia University Medical Center’s Institutional Animal Care and Use Committee (protocol #: AC-AAAD4453). Studies were conducted in age-matched, 3-month old, male C57BL/6 mice (Jackson Laboratory, Bar Harbor, ME, USA) unless otherwise stated. Some experiments were also carried out in wild type (WT) and *Cyp2e1*^*-/-*^ mice in a mixed genetic background, the generation of which has previously been described [16]. All mice were maintained in the Columbia University Medical Center animal facility using standard caging with environmental enrichment (hiding structures, and bedding and nesting material) and climate controlled environmental conditions. Mice were monitored daily and weighed weekly. We have employed methods we reported previously for feeding mice alcohol [13, 17–21], with a detailed methodological description found elsewhere [20]. Briefly, in this study we used the high-fat formulation of the Lieber-DeCarli liquid diet, which contained 4 IU vitamin A/g (Bio-Serv, Frenchtown, NJ, USA) [21]. The macronutrient composition of these diets was as follows, Control diet: protein 150, fat 360, carbohydrates 490; Alcohol diet: protein 150, fat 360, carbohydrates 135, ethanol 355 (All units Kcal/L). At the start of the alcohol feeding protocol, mice were transferred into individual cages and randomly assigned to an experimental group. All experimental mice underwent a one-week acclimation period consuming an alcohol-free control diet. Mice in the alcohol group then went through an adaptation period consisting of one week consuming 2.1% v/v alcohol and one week consuming 4.2% v/v alcohol. This was followed by a 2-week period where mice in the alcohol group were fed the liquid diet containing 6.4% v/v alcohol. Control mice were fed the alcohol-free control diet throughout the entire alcohol feeding protocol and were pair-fed an isocaloric volume of liquid diet determined from the volume the mice in the alcohol group consumed in the preceding 48 h. At the end of the alcohol feeding protocol, mice underwent carbon dioxide euthanasia, and the liver was immediately collected, a portion of which was reserved for histology and the remainder snap frozen in liquid nitrogen and stored at -80°C until analysis.

### Measurement of hepatic triglycerides and retinoid content

Hepatic steatosis was assessed using histological and biochemical approaches. Neutral lipid accumulation in the liver was visualized by staining with Oil Red O, following tissue preparation by the Columbia University Medical Centre’s Molecular Pathology core facility. Images of stained livers were collected using an FSX100 microscope (Olympus, Center Valley, PA, USA). To measure hepatic triglycerides (TG), total hepatic lipids were extracted using a Folch solution [22], and the concentration of TG measured using an Infinity Triglycerides liquid stable reagent (Thermo-Fisher Scientific; Middleton, VA). The measurement of hepatic retinol and retinyl ester levels was performed using standard HPLC methods [23], with a 4.6 × 260 mm Waters Symmetry C18 column (Waters Corp., Milford, MA, USA). The concentrations of retinol and retinyl ester were calculated using the area under the curve of chromatogram peaks (λ = 325 nm) and corrected to the amount of recovered retinyl acetate internal standard (Sigma-Aldrich, St Louis, MO, USA). A Xevo TQ MS Acquity UPLC system (Waters) was used to measure hepatic RA levels as previously described [24].

### Quantitative PCR

The hepatic mRNA expression level of genes of interest was determined by real-time quantitative PCR (qPCR) using standard methods. In brief, total RNA was extracted from liver samples using TRIzol (Invitrogen, Carlsbad, CA), and cleaned up using a RNeasy column (Qiagen, Valencia, CA). Purified RNA (2 μg) was reverse transcribed into cDNA using a high-capacity cDNA RT Kit according to the manufacturer’s instructions (Applied Biosystems, Carlsbad, CA). All qPCR amplification was performed under uniform reaction conditions using a LightCycler480 real-time PCR cycler (Roche Diagnostics, Indianapolis, IN, USA). To analyze qPCR data, threshold cycles were calculated for target genes and compared to the reference gene *Actb*, as previously described [25]. The following gene specific primers were used to amplify targets of interest: *Actb* (Forward primer: 5′-AGC TAT GAG CTG CCT GAC G-3′; Reverse primer: 5′-TGC CAC AGG ATT CCA TAC CCA AG-3′; amplicon = 73 bp)*; Cyp26a1* (Forward primer: 5′-GGC ACT GTG ATT GGC AGC TTC TAA-3′; Reverse primer: 5′-TGC AGG ATT GTC CAC AGG GTA-3′; amplicon = 73 bp), *Cyp26b1* (Forward primer: 5′-GCA GTA TAT GCT TAT GAC ATC TGA ATC-3′; Reverse primer: 5′-CCT GAC CAC TCA CCA ACA AA-3′; amplicon = 77 bp), *Cyp2c29* (Forward primer: 5′-TCT GGC AAG CAC TAT CAA TGA CCT-3′; Reverse primer: 5′-GGA CTT TAG CTG TGA CAT CTG GG-3′; amplicon = 113 bp), *Cyp2c39* (Forward primer: 5′-CTG ATA GAG GAA GCA TTC CAA TGG T-3′; Reverse primer: 5′-TCG TGA GTG TGA AGC GCC-3′; amplicon = 113 bp), *Cyp3a11* (Forward primer: 5′-AAG ACA AAG TCT CTC ATA AAG CCC T-3′; Reverse primer: 5′-GGA AAG TGT GCT ACT GGT GGT T-3′; amplicon = 101 bp).

### Statistical analysis and sample size

**Fig 1:**
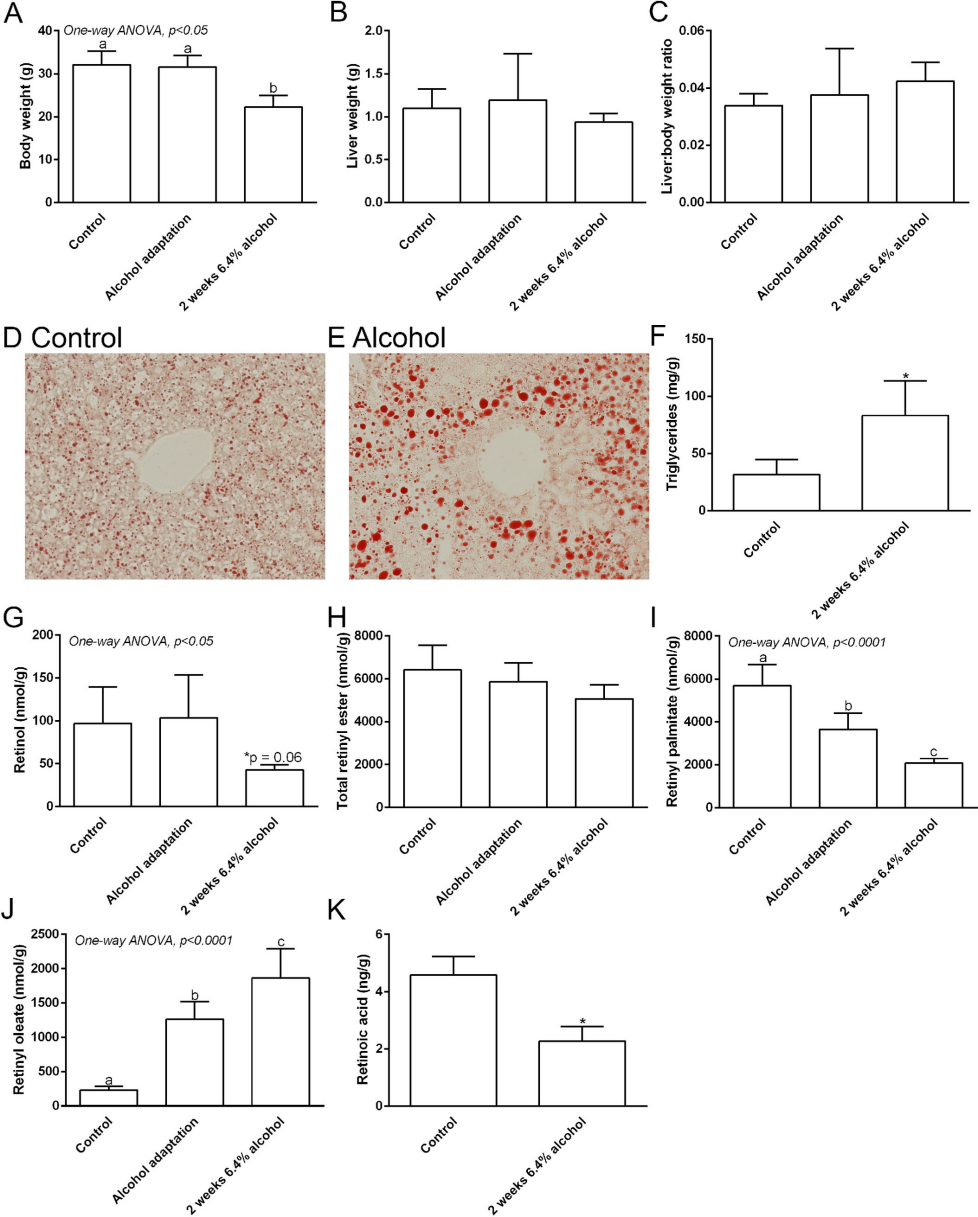
Chronic alcohol consumption depletes hepatic retinoid content. Body weight decreases in the 2-week 6.4% alcohol fed mice (A), but there was no significant change in the liver weight (B) or liver to body weight ratio (C). Representative images of Oil Red O Staining of liver in control (D) and alcohol fed mice (E) shows more lipid droplets in alcohol-fed mice. Chronic alcohol consumption is associated with a significant increase in hepatic triglyceride content (F). Hepatic retinol level is significantly decreased following alcohol adaptation (G). Total hepatic retinyl ester levels are not significantly altered following alcohol adaptation (H). The hepatic level of retinyl palmitate is significantly decreased following alcohol adaptation (I). A significant increase in hepatic retinyl oleate level is associated with chronic alcohol consumption (J). The hepatic retinoic acid level is significantly decreased following chronic alcohol consumption (K). * p <0.05 *vs*. control; Student’s *t*-test (F and K). Columns with different lowercase letters indicate significant difference; One-way ANOVA (A-C and G-J).

**Fig 2:**
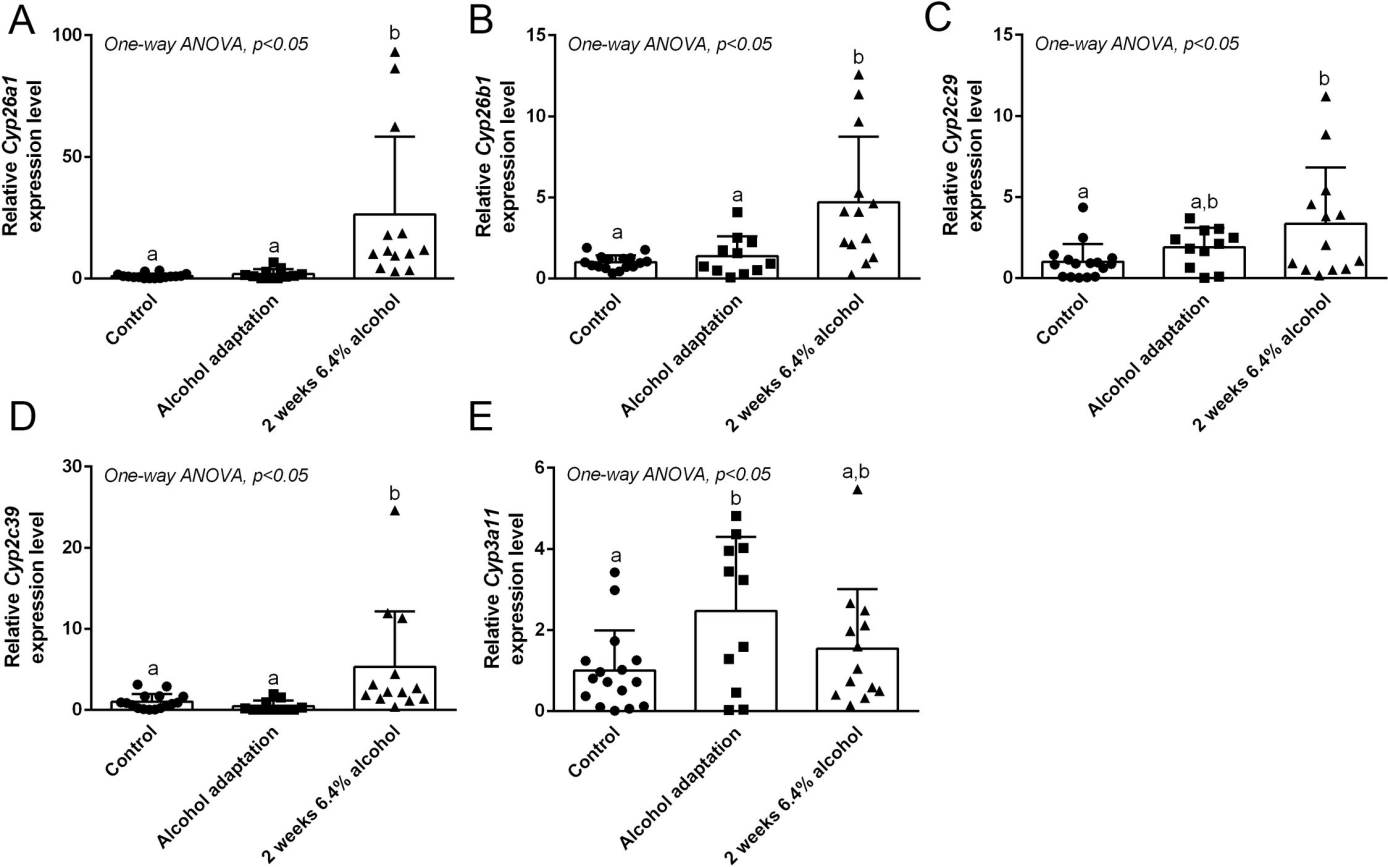
Chronic alcohol consumption induces multiple retinoid-catabolizing CYP enzymes. Effect of chronic alcohol consumption on hepatic mRNA expression level of *Cyp* enzymes are presented as follows: *Cyp26a1* (A), *Cyp26b1* (B), *Cyp2c29* (C), *Cyp2c39* (D), and *Cyp3a11* (E). Columns with different lowercase letters indicates significant difference; One-way ANOVA; *P <0.05 *vs*. control for (A-E).

**Fig 3:**
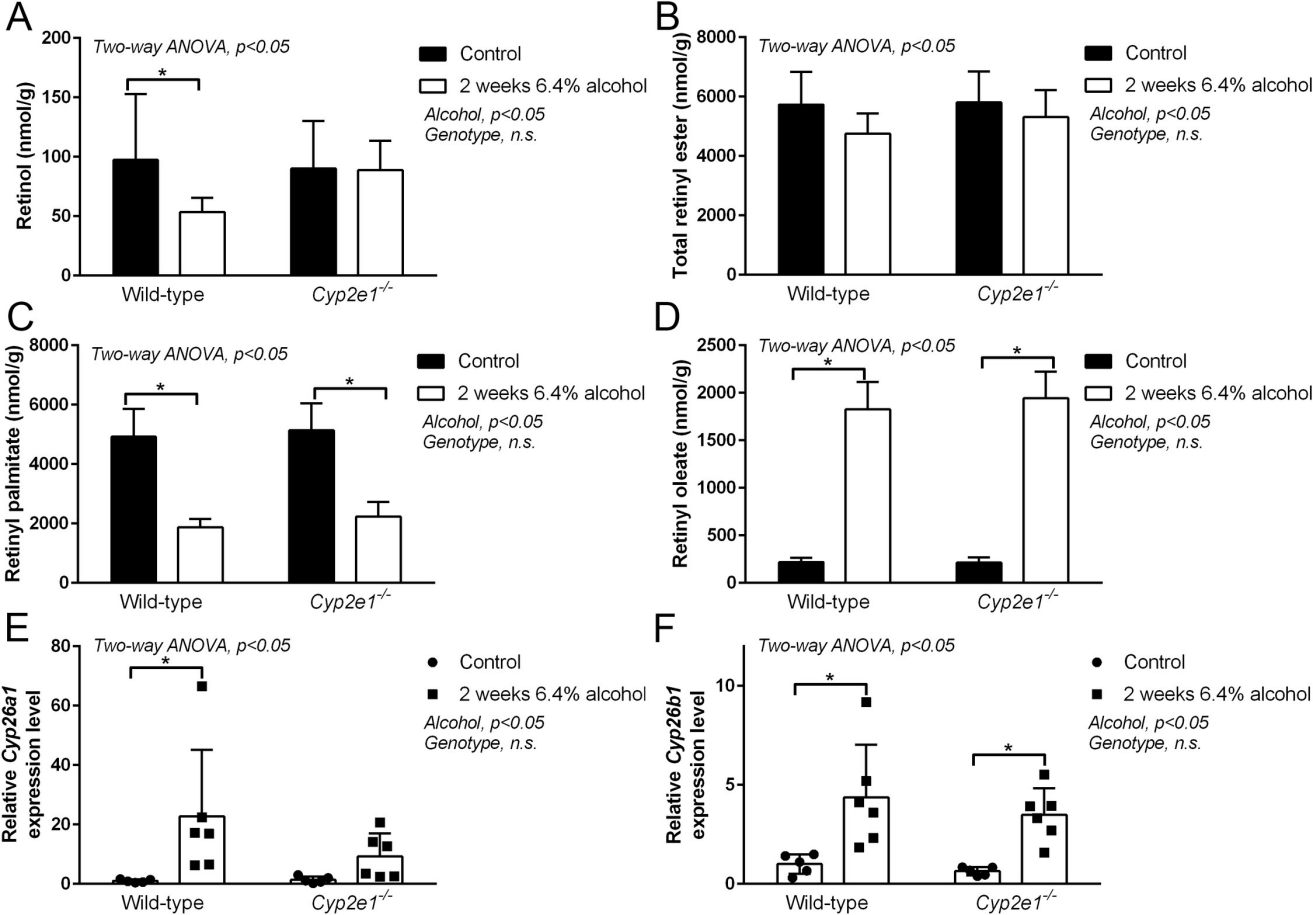
Effect of genetic ablation of CYP2E1 on alcohol induced hepatic retinoid metabolism. Consumption of 6.4% alcohol for 2 weeks significantly decreased hepatic retinol level in wild type (WT) mice but did not affect retinol levels in *Cyp2e1*^*-/-*^ mice (A). Total hepatic retinyl ester levels are not significantly different between WT and *Cyp2e1*^*-/-*^ mice (B). Consumption of 6.4% alcohol for 2 weeks significantly decreased hepatic retinyl palmitate levels in both WT mice and *Cyp2e1*^*-/-*^ mice (C). Consumption of 6.4% alcohol for 2 weeks significantly increased hepatic retinyl oleate level in both WT mice and *Cyp2e1*^*-/-*^ mice (D). Consumption of 6.4% alcohol for 2 weeks significantly increased *Cyp26a1* expression levels in the WT mice not in the *Cyp2e1*^*-/-*^ mice (E). Consumption of 6.4% alcohol for 2 weeks significantly increased *Cyp26b1* expression level both in the WT and *Cyp2e1*^*-/-*^ mice (F). Data analyzed by Two-way ANOVA; *P <0.05 *vs*. control for Tukey’s multiple comparison post-test (A-F).

The results presented in this manuscript were obtained by analyzing unpublished data from multiple independent alcohol feeding studies. The phenotypic description of our alcohol feeding study on hepatic lipid accumulation (Fig 1A–1F) was taken from a single alcohol feeding study: control n = 12, alcohol n = 6. Our HPLC data describing the effect of chronic alcohol consumption on hepatic retinoid levels (Fig 1G–1J) were taken from a single chronic alcohol feeding study: control n = 7, alcohol adaptation n = 6, and 2 weeks 6.4% alcohol n = 5. Measurement of hepatic retinoic acid levels by mass spectrometry (Fig 1K) were pooled from two independent alcohol feeding studies with consistent results: control n = 9, alcohol n = 12. Gene expression data presented in Fig 2 is pooled from two independent alcohol feeding studies with consistent results: control n = 16, alcohol adaptation n = 11, and 2 weeks 6.4% alcohol n = 13). HPLC data from wild-type and alcohol consuming *Cyp2e1*^*-/-*^ mice (Fig 3A–3D) were pooled from two independent alcohol feeding studies with consistent results: wild-type control n = 10, wild-type alcohol n = 11, *Cyp2e1*^*-/-*^ control n = 10, *Cyp2e1*^*-/-*^ alcohol n = 13. Gene expression data from wild-type and alcohol consuming *Cyp2e1*^*-/-*^ mice (Fig 3E and 3F) were from a single alcohol feeding study: wild-type control n = 5, wild-type alcohol n = 6, *Cyp2e1*^*-/-*^ control n = 5, *Cyp2e1*^*-/-*^ alcohol n = 6.

All numerical data were compiled in Excel (Microsoft, Redmond, WA) and analyzed using Prism 6 (GraphPad Software, La Jolla, CA, USA). Data are presented as the mean ± standard deviation (SD). When comparing experimental data with two groups, a Student’s t-test was used. When comparing experimental data with three groups, a one-way ANOVA was used with Tukey’s multiple comparisons post-test. When comparing experimental data with four groups, a Two-way ANOVA was used with Tukey’s multiple comparisons post-test. For all statistical analyses, a p-value <0.05 was considered statistically significant.

## Results

### Chronic alcohol consumption induces hepatic steatosis and depletes hepatic retinoids

To examine the effects of chronic alcohol consumption on hepatic TG and retinoid levels, liver tissues were collected from control and alcohol-fed WT mice. Body weight of the 6.4% alcohol fed group was reduced compared to control and adaptation group (Fig 1A). However, there was no significant difference in the liver weight (Fig 1B) or liver to body weight ratio (Fig 1C) among the groups. Oil Red O staining of liver tissue revealed more staining in the livers of alcohol-fed mice after two weeks of consuming 6.4% alcohol. This is indicative of increased hepatic lipid accumulation (Fig 1D and 1E). This observation was confirmed by quantitative measurement of hepatic TG content, showing significantly higher levels of hepatic TGs in alcohol-fed mice (Fig 1F; Student’s t-test p <0.05). These data show that our alcohol feeding protocol successfully induced hepatic steatosis in mice. Next, we sought to determine alcohol’s effect on hepatic retinoid levels, focusing on changes after the alcohol adaptation period, and after two weeks of consuming 6.4% alcohol. In agreement with our previously published observations [13, 20], hepatic retinol levels decreased in alcohol-consuming mice (Fig 1G; One-way ANOVA p <0.05), with a trend for a decrease in the control vs. two-week time point (Fig 1G; post-test p = 0.06). Total hepatic retinyl esters did not significantly change in mice fed alcohol (Fig 1H; One-way ANOVA p = 0.09). However, when we analyzed the concentration of specific retinyl esters, we observed that retinyl palmitate levels decreased significantly (Fig 1I; One-way ANOVA p <0.0001), with a compensatory increase in retinyl oleate (Fig 1J; One-way ANOVA p <0.0001). RA is one of the active metabolites of vitamin A and participates in hepatic retinoid signaling, therefore we also measured its concentration in the experimental mice [6]. Our results show that hepatic RA levels were also decreased in mice consuming 6.4% alcohol for 2 weeks, compared to control mice (Fig 1K; Student’s t-test p <0.05). Taken together, these data reveal that the alcohol feeding protocol induced significant hepatic steatosis and depleted hepatic retinoid content.

### Chronic alcohol consumption induces multiple retinoid catabolizing CYP enzymes

Our data show that alcohol consumption leads to decreased hepatic retinoid levels, including a lowered hepatic RA concentration. It has been suggested that alcohol-induced hepatic retinoid depletion is associated with hepatic CYP2E1-mediated retinoid catabolism [14], while we and others have also explored the potential contribution of other hepatic CYPs [13, 26]. Here, we sought to establish whether alcohol can induce the expression of retinoid-catabolizing CYPs at the gene and protein expression level (Fig 2), possibly accounting for the diminished tissue RA levels we observed. To address this, we explored the expression of the RA-specific hydroxylases CYP26A1 and CYP26B1, as well as CYP2C29, CYP2C39, and CYP3A11, which are broader-specificity CYP enzymes with the capability to metabolize RA [15]. Our results show that the relative expression of *Cyp26a1* is significantly increased, with ~20-fold higher expression in mice fed 6.4% alcohol for 2 weeks compared to the control and adaptation group (Fig 2A; One-way ANOVA p <0.05). Relative expression of *Cyp26b1* is also significantly increased (~4-fold) in mice fed 6.4% alcohol for 2 weeks compared to the control and adaptation group (Fig 2B; One-way ANOVA p <0.05). Thus, chronic alcohol consumption increases the gene expression levels of the RA-specific hydroxylases *Cyp26a1* and *Cyp26b1*. The relative expression of *Cyp2c29* was significantly higher in mice fed 6.4% alcohol for 2 weeks (~3-fold) compared to control but no significant difference was observed in the adaptation group (Fig 2C; One-way ANOVA p <0.05). The relative expression of *Cyp2c39* was significantly increased (~5-fold) in mice fed 6.4% alcohol for 2 weeks compared to the control and adaptation group (Fig 2D; One-way ANOVA p <0.05). Expression of *Cyp3a11* was relatively higher in the adaptation group versus control, but not significantly different between mice fed 6.4% alcohol for 2 weeks (Fig 2E; One-way ANOVA p <0.05). As expected, the gene expression level of *Cyp2e1* was not significantly different in the control vs. alcohol fed mice (data not shown). Taken together, our results show that chronic alcohol consumption induces mRNA expression of multiple retinoid-catabolizing CYP enzymes.

### Effect of genetic ablation of CYP2E1 on alcohol-induced hepatic retinoid depletion

As discussed above, CYP2E1 had been hypothesized to mediate degradation of hepatic retinoids when induced by chronic alcohol consumption [5, 11, 14]. Thus, the effects of alcohol consumption on hepatic retinoid metabolism in *Cyp2e1*^*-/-*^ mice provide an opportunity to perform a genetic dissection of CYP2E1’s contribution to hepatic retinoid depletion. Here, we confirm and extend our previous observations in *Cyp2e1*^*-/-*^ mice [13], and show that hepatic retinol content is significantly lower in WT mice fed alcohol, whereas the alcohol-induced decline in hepatic retinol content is not seen in *Cyp2e1*^*-/-*^ mice, compared to control mice (Fig 3A; Two-way ANOVA p <0.05). In contrast to the effect of *Cyp2e1*-deficiency on hepatic retinol levels, total hepatic retinyl ester levels were decreased to a similar extent in both WT and *Cyp2e1*^*-/-*^ mice fed-alcohol (Fig 3B; Two-way ANOVA p <0.05). Here, we extend this observation to show that alcohol-induced changes in hepatic retinyl palmitate (Fig 3C; Two-way ANOVA p <0.05) and retinyl oleate (Fig 3D; Two-way ANOVA p <0.05) levels are not different between WT and *Cyp2e1*^*-/-*^ mice. Alcohol consumption significantly depleted hepatic retinyl palmitate levels to the same extent in WT and *Cyp2e1*^*-/-*^ mice (Fig 3C). Similarly, the alcohol-associated compensatory increase in hepatic retinyl oleate levels was comparable in WT and *Cyp2e1*^*-/-*^ mice (Fig 3D). Taken together, this data confirms the proposal that CYP2E1 has a role in alcohol-induced retinol depletion, but not retinyl ester depletion.

To better understand the relationship between CYP2E1 induction and the depletion of hepatic retinoids, we studied the gene expression levels of CYP26A1 and CYP26B1 in WT and *Cyp2e1*^*-/-*^ mice. Specifically, we wanted to determine whether the induction of these RA catabolizing CYPs was blunted in *Cyp2e1*^*-/-*^ mice, as has been shown for CYP2A5 *in vivo* [27]. Consistent with our results from above, relative expression levels of both *Cyp26a1* and *Cyp26b1* are significantly increased in mice fed alcohol (Fig 3E and 3F, respectively; Two-way ANOVA p <0.05). In WT mice, the post-test for change in expression of *Cyp26a1* was significant, whereas in *Cyp2e1*^*-/-*^ mice, the post-test for the change in expression level of *Cyp26a1* was not. Our post-test analysis showed that *Cyp26b1* was significantly increased in both WT and *Cyp2e1*^*-/-*^ mice fed alcohol. These data suggest that in the absence of CYP2E1, RA-catabolizing CYPs are still induced.

## Discussion

The aim of this study was to gain a better understanding of the involvement of CYP enzymes in the alcohol induced depletion of hepatic retinoids. We sought to test the hypothesis that alcohol induced depletion of hepatic retinoid is due to CYP-mediated oxidative metabolism by surveying the expression of known retinoid-catabolizing CYP enzymes in alcohol fed mice, and dissecting out the contribution of CYP2E1 to alcohol induced hepatic retinoid depletion.

Consistent with the well-described application of Lieber-DeCarli liquid diets, we show that our alcohol feeding protocol successfully induced hepatic steatosis in mice, as evidenced by the accumulation of hepatic lipid droplets and TGs [28]. In agreement with our previous studies [13, 20], alcohol consumption also decreased hepatic retinoid levels and changed the acyl composition of hepatic retinyl esters. Here, we extend our past observations showing alcohol-induced hepatic retinol and retinyl ester depletion and show that chronic alcohol consumption is also associated with a decrease in hepatic RA levels, as measured by mass spectrometry. Interestingly, it has been reported that RA levels are unchanged in mice chronically consuming alcohol, also using mass spectrometry [26], but our result for the experimental protocol we employed is consistent with older literature showing that alcohol consumption decreases RA in hepatic and extrahepatic tissues, as measured by HPLC [12, 13, 29, 30]. We are confident in the conclusion that chronic alcohol consumption leads to the depletion of hepatic retinol, retinyl esters, and RA.

When considering the mechanism for alcohol-induced hepatic retinoid depletion, it has been proposed that alcohol-inducible hepatic CYP enzymes are major contributors [12, 13, 31]. As recently reviewed, it was proposed that CYP2E1 is a major contributor to alcohol-induced hepatic retinoid degradation [32]. However, we and others have considered the role of other CYP enzymes, including the RA-specific hydroxylases CYP26A1 and CYP26B1, as well as other CYP enzymes with broader substrate affinities that are known to catabolize RA [26]. In this study, we observed significantly higher mRNA expression levels of *Cyp26a1* (~20 fold) and *Cyp26b1* (~4 fold) in the liver of alcohol-fed mice compared to control. Other broader substrate affinity CYP enzymes are also induced by alcohol consumption, including *Cyp2c29* (~3 fold), *Cyp2c39* (~5 fold) and *Cyp3a11* (~1.5 fold), though the level of mRNA induction is not as great as we observed for *Cyp26a1*. In addition to CYP2E1, our data provides evidence that chronic alcohol consumption leads to the induction of two specific retinoid-catabolizing CYP enzymes CYP26A1 and CYP26B1, and suggests that these CYPs also contribute to the loss of hepatic retinoid levels and the pathogenesis of ALD [33].

Given the prominent role that CYP2E1 has been proposed to play in alcohol-induced hepatic retinoid depletion [11, 14, 33], we performed a genetic dissection of CYP2E1’s contribution in alcohol-fed *Cyp2e1*^*-/-*^ mice. We found that the depletion of hepatic retinol content is blunted in alcohol-fed *Cyp2e1*^*-/-*^ mice, but alcohol’s effect on hepatic retinyl ester levels and acyl composition is not different between *Cyp2e1*^*-/-*^ mice and WT mice. These differential effects of CYP2E1 ablation may reflect the major cell type where hepatic retinoids are found and where CYP2E1 is expressed. In our HPLC data, retinol primarily reflects the retinoid pool in hepatocytes, and retinyl esters reflect the retinoid pool in HSCs [34]. CYP2E1 is primarily known to be expressed in hepatocytes, and not HSCs [35]. Thus, our data fits a model whereby CYP2E1 contributes to hepatocyte retinol depletion but has no effect on HSC retinyl ester stores. Given that HSCs store 90–95% of hepatic retinoids in the form of retinyl ester [34], this difference is physiologically significant and raises the question of what other CYPs may be involved. In this context, it is interesting to note that CYP26B1, which we show is induced by alcohol, is specifically expressed in HSCs, whereas CYP26A1 is specifically expressed in hepatocytes [36]. Moreover, we report here for the first time that alcohol-induced changes in hepatic retinyl ester acyl composition are independent of CYP2E1, which again likely reflects the predominant expression of CYP2E1 in hepatocytes and the fact that hepatic retinyl esters are primarily stored in HSCs. Other than serving as a sensitive marker of alcohol consumption, the pathophysiological significance of altered retinyl ester acyl composition, and the molecular basis for this change, requires further study [20].

*Cyp2e1*^*-/-*^ mice are protected from the alcohol-induced depletion of hepatic retinol. It has previously been shown that the induction of specific CYPs may be dependent on CYP2E1 [13, 20]. For example, it has been shown that the ethanol induction of CYP2A5 only occurs after the induction CYP2E1 [27]. Thus, it is possible that the blunted depletion of hepatic retinol by alcohol in *Cyp2e1*^*-/-*^ mice may not be related to the direct loss of CYP2E1 *per se*, but rather the failure of other retinoid-catabolizing CYPs to be induced in the absence of CYP2E1. Interestingly, the induction of *Cyp26a1 (~5-fold)* in *Cyp2e1*^*-/-*^ mice after alcohol consumption did not reach statistical significance, as compared to the significant induction in WT mice (~15-*fold*). On the other hand, *Cyp26b1* is significantly increased in both WT and *Cyp2e1*^*-/-*^ mice.

While our data provide evidence for ethanol-induced expression of retinoid-catabolizing CYPs, the mechanism for this remains unclear and requires further study. Depletion of hepatic retinoids is likely multi-factorial and cell-type dependent. As discussed above, as part of the microsomal ethanol oxidizing system, the induction of CYP2E1 appears to play a role in retinoid depletion in hepatocytes, but our data shows that the induction of CYP26A1 and CYP26B1 is not dependent on CYP2E1. CYP26A1 and CYP26B1 induction in alcohol consuming mice may be secondary to the broad induction of hepatic CYPs and the microsomal ethanol oxidizing system upon chronic ethanol exposure, or a response to specific changes in hepatic retinoid metabolism. Interestingly, CYP2E1 has been shown to catabolize retinol into polar retinoid metabolites which are cytotoxic to hepatocytes and induce oxidative stress [11, 37]; moreover, many of these retinoid metabolites are potent inducers of CYP26A1 and undergo further catabolism by CYP26A1 as observed *in vitro* [38]. This is one possible mechanism to explain hepatic CYP26 induction in alcohol consuming mice, but the elucidation of this mechanism and other possible contributors requires further study.

We recognize several limitations in this study. Our conclusions are primarily based on observed changes in gene expression levels and the concentration of hepatic retinoids (retinyl esters, retinol, and RA). We did not quantitatively measure CYP protein expression levels or enzyme activity in our experiments. Hepatic retinol and retinyl ester levels were routinely measured by HPLC in all experiments; however, we only used the technically more challenging mass spectrometry method to measure hepatic RA concentrations in one experiment. As discussed above, the mechanism underlying the induction of retinoid catabolizing CYPs is unclear and is the focus of ongoing studies.

## Conclusions

Chronic alcohol consumption in mice decreases hepatic RA levels in addition to the well-established depletion of retinol and retinyl ester. The alcohol-induced depletion of hepatic retinoids is associated with a concurrent increase in multiple RA-catabolizing CYPs, including most prominently CYP26A1 and CYP26B1. CYP2E1 appears to be a contributor to retinoid depletion in hepatocytes, but not HSCs. Future research into the liver cell type-specific induction of different retinoid catabolizing CYPs may provide further insight into the pathogenesis of ALD.
